# Molecular Evaluation of Expression Changes in Genes Associated With Colistin Resistance and Virulence Development in *Salmonella enterica* From Two Iran Hospitals

**DOI:** 10.1155/cjid/2926422

**Published:** 2025-10-10

**Authors:** Mohammad Darvishi, Shahriar Sepahvand, Hassan Sepahvand, Mohammad Ali Davarpanah, Mahboobeh Madani, Hesam Kamyab, Seyed Sobhan Behrouz, Farzaneh Asmani, Simin Yazdanpanah Ravari

**Affiliations:** ^1^Department of Aerospace & Subaquatic Medicine, Infectious Diseases & Tropical Medicine Research Center, AJA University of Medical Sciences, Tehran, Iran; ^2^Department of Microbiology, Fal.C., Islamic Azad University, Isfahan, Iran; ^3^Department of Medical, The I.M. Sechenov First Moscow State Medical University (Sechenov University), Moscow, Russia; ^4^HIV/AIDS Research Center, Institute of Health, Shiraz University of Medical Sciences, Shiraz, Iran; ^5^Department of Biomaterials, Saveetha Dental College and Hospital, Saveetha Institute of Medical and Technical Sciences, Chennai 600077, India; ^6^The KU-KIST Graduate School of Energy and Environment, Korea University, 145 Anam-ro, Seongbuk-Gu, Seoul 02841, Republic of Korea; ^7^Department of Biotechnology, Faculty of Chemistry, University of Kashan, Kashan, Iran; ^8^Department of Biology, SR.C., Islamic Azad University, Tehran, Iran

**Keywords:** colistin, fimbriae, flagellin, genes expression, real-time PCR, *Salmonella enterica*

## Abstract

*Salmonella enterica*is one of the most frequent causes of gastroenteritis in humans. The emergence of antimicrobial-resistant strains of the bacterium, especially those which are resistant toward colistin (CST), the latest antibiotic introduced to cure this bacterial disease, has become a severe health problem. Beside *pmrA* and *pmrB* genes, CST resistance is determined by mobilized colistin resistance (*mcr*) genes. Accordingly, in the present study, the expression pattern of these genes and *fliC* (encoding the flagellin protein) and *agfA* (encoding the bacterial fimbriae) was evaluated. To this end, a total of 50 *S. enterica* isolates were collected from two hospitals in Shiraz, Iran, during 2019-2020. The pattern of antimicrobial resistance of the isolates was determined by the disk diffusion method. Then, after the antibiotic sensitivity test, following RNA extraction, the expression of selected genes was evaluated running real-time PCR. Results revealed that the occurrence of CST resistance in Salmonella isolates was 16%. The transcription levels of *pmrA* and *fliC* genes were increased in CST-resistant isolates. The results demonstrated the widespread distribution of multidrug-resistant and CST-resistant strains of *S. enterica* in the hospital setting. To overcome this issue, further actions, such as fast detection and eradication and appropriate preventive measures, should be undertaken to face this challenge.

## 1. Introduction

The genus *Salmonella* belongs to the Enterobacteriaceae family [1]. The bacteria of this genus are Gram-negative bacilli, most of which are motile. Bacteria belonging to genus *Salmonella* could be both aerobic and anaerobic with optimal growth temperature of 37°C [2]. There are over 2650 serotypes of *Salmonella*, some of which are important enteric pathogens [3]. *Salmonella* species can infect humans and animals like chickens, turkeys, cows, and pigs. These species cause gastrointestinal disorders and are considered among the most important foodborne pathogens [4] and the main causative agents of the etiology of severe systemic diseases such as typhoid and paratyphoid fevers [5].

Virulence factors in *Salmonella* are divided into two major categories based on their genetic control: chromosomal and plasmid virulence factors. The importance of flagellum is that it allows movement, chemotaxis, and the establishment of bacteria in tissues. Flagellin also stimulates the immune system via the toll-like receptor 5 (TLR5) [6–8]. *FliC*, together with *FljB*, represents the structural components of the *Salmonella* flagellum highly expressed on the cell surface and regulated by transcription repressor *FljA* [9–12]. Fimbriae play a critical role in virulence by allowing bacteria to interact with host cells and other solid substrates [13]. Aggressive factor A (*agfA*) gene is also involved as a gene contributing to the production of fimbriae in the bacterial colonization in the intestine and systemic infection in the host [14].

The emergence of multidrug-resistant (MDR) bacterial strains has become a significant challenge for the health community [15] that can affect patients, economics, and the health system. It has considerable implications on public health. MDR is associated with prolonged length of stay, increased cost, and mortality [16]. Various bacterial pathogens have gained the ability to resist different antimicrobials. MDR *Salmonella* strains have evolved since the 1980s, and many salmonellosis outbreaks have been reported worldwide, especially from developing countries [17–19]. This issue becomes worse when some strains become resistant to new generations of antimicrobials or old efficient antibiotics. Colistin (CST) is an old antibiotic (over 50 years old) that is efficient against MDR Gram-negative bacteria, including *Salmonella* [20]. It should be noted that it is used as a last-line therapy against infections caused by these bacteria and is considered an antibiotic of critical importance in human clinical settings by the World Health Organization (WHO) [21]. However, due to the excessive consumption of this antibiotic by humans and the widespread use of this antibiotic in the veterinary medicine and livestock/animal farming [22], CST resistance is increasing among Gram-negative bacteria, including *Pseudomonas aeruginosa*, *Acinetobacter baumannii*, *Escherichia coli*, and *S. enterica* [21, 23].

As an antibiotic, CST targets lipid A moiety of the lipopolysaccharide (LPS), which is a surface antigen located in the outer membrane (OM) of Gram-negative bacteria [24]. LPS plays an essential role in the manipulation of OM permeability [25]. Through electrostatic interactions, positively charged moieties of CST (diaminobutyric acid residues) bind to lipid A. These interactions destabilize LPS and make the OM more porous. As a result, transportation via OM is disrupted, which will, in turn, lead to cell death.

By reducing the negative charge of LPS, mainly by the addition of positively-charged molecules such as phosphoethanolamine (PEtn) or 4-amino-4-deoxy-L-arabinose (L-Ara4N), the interaction between LPS and CST will be reduced; hence, the cell permeability will not be disrupted, and the bacteria will survive [26]. CST resistance in *Salmonella* is mainly chromosomal; however, plasmids might play a role in this phenomenon. In chromosomal resistance, two different regulatory systems, PmrA/PmrB and PhoP/PhoQ, make a significant contribution in this regard. PmrA/PmrB is involved in the synthesis of L-Ara4N, while PhoP/PhoQ is responsible for the biosynthesis of PEtn [27]. Specific mutations in the *Salmonella* genome led to the permanent expression of the components of these two systems. The addition of positively charged molecules, L-Ara4N and PEtn, to lipid A makes the CST–lipid A interaction less favorable. In addition, other strategies destabilize the CST–lipid A interaction, such as PagL-mediated lipid A deacylation [28]. The mobilized colistin resistance (*mcr*)*-1* gene was mostly detected in *Salmonella*, but *mcr-2*, *mcr-3*, *mcr-4*, *mcr-5*, and *mcr-9* were also detected. Since it has been reported for other bacteria, the direct relationship between the presence of *mcr* genes and CST resistance in *Salmonella* has not been conclusively established. These genes have also been found in sensitive strains [29].

In the present study, the presence and expression of two resistance related genes, i.e., *pmrA* and *pmrB*, and two virulence genes encoding fimbriae and flagellin proteins, *agfA* and *fliC* genes, respectively, were evaluated in patients with salmonellosis.

## 2. Materials and Methods

### 2.1. Informed Consent

All human participants consciously participated in this study, and sampling was performed with the patients' complete satisfaction. The study samples were collected from two hospitals in Shiraz, Iran: Ali Asghar Hospital and Ghadir Mother and Child Subspecialized Hospital during 2019 and 2020.

### 2.2. Isolation and Identification of Bacterial Strains

This cross-sectional descriptive study was conducted on patients suspected of infection with *Salmonella enterica*. According to the standard method, clinical specimens, including stool, blood, body fluids, etc., were taken from the suspected cases of salmonellosis infection. After sampling, the stool samples were immediately transferred to the selenite F medium for the selective enrichment of *Salmonella* species. The samples were incubated at 24°C for 6 hours. Then, the bacteria were transferred to selective culture media, such as Salmonella–Shigella (SS) agar, sulfite agar Bismuth, and other media and incubated at 37°C for 24 h. Blood samples were first cultured in the biphasic (solid and liquid) medium and then transferred to the selective culture media. The following day, *Salmonella*-suspected colonies were isolated and identified using biochemical tests. According to the manufacturer's instructions, colonies were further confirmed using a Microgen microbial identification kit (Microgen, UK).

### 2.3. Antibiotic Susceptibility Testing

Antibiogram test was performed using the disc agar-diffusion method [30] (in order to investigate the susceptibility of the isolates to different antibiotics (MAST UK Company), including tetracycline (TET), nalidixic acid (NAL), cefazolin (CFZ), ampicillin (AMP), chloramphenicol (CHL), and CST. *Salmonella enterica* subsp. enterica serovar Enteritidis ATCC 13076 strain was used as the positive control. The inhibition (nongrowing) zones were measured using a ruler and compared with existing Clinical and Laboratory Standards Institute (CLSI) tables for different antibiotics.

### 2.4. DNA Extraction

For DNA extraction, each isolate was grown in Luria Bertani broth (LBH). After 16 h of incubation at 37°C, DNA extraction and purification were performed using a commercial kit (CinnaGen, Iran) according to the manufacturer's instructions.

### 2.5. Tracking the *hilA* Gene via Polymerase Chain Reaction (PCR)

A pair of specific primers was used to identify the *hilA* gene. The forward primer sequence was 5′-CGG AAG CTT ATT TGC GCC ATG CTG AGG TAG-3′ and the reverse primer sequence was 5′-GCA TGG ATC CCC GCC GGC GAG ATT GTG-3′.

### 2.6. Amplification of the Selected Genes

PCR was used to amplify *pmrA*, *pmrB*, *fliC*, and *agfA* genes. The quality of the designed/applied primers in this study (Table 1) was checked on the NCBI web (https://www.ncbi.nlm.nih.gov/tools/primer-blast/). The primers' sequences and source are illustrated in Table 1. PCR amplification was performed in a total reaction volume of 25 μL containing 2.5 μL of 10 × buffer (Fermentas, Canada), 250 μM of each primer, 0.2 mM of each dNTP, 2.5-mM MgCl2, 0.75 U of Taq DNA polymerase ((Fermentas, Canada), 2 ng of purified DNA (as the template), and variable distilled water. PCR cycle condition was as follows: 94°C for 2 min; 35 cycles of 93°C for 1 min (denaturation step); 50°C–60°C for 1 min (annealing step); 72°C for 1 min (extension step); and 72°C for 10 min. After performing PCR, 5 μL of each reaction product was run on a 1% agarose gel, and the results were recorded using BioDoc Analyzer (Biometra, Germany).

### 2.7. RNA Extraction

RNA extraction was performed using bacterial RNA Isolation Kit Trizol Max (Invitrogen Life Technologies) following the provided protocol. Bacterial cells were harvested, resuspended in 200-μL accelerating factor, and incubated at 95°C for 4 min. After that, the cells were lysed in 1-mL Trizol. The lysed cells were incubated in the Trizol for 5 min at room temperature. A total of 100 μL of chloroform was added to the mixture and strongly shaken for 15 s. The mixture was incubated at room temperature for 2-3 min. The phases were separated by centrifugation at 12, 000 × g for 15 min, and 90% of the liquid phase (transparent color) was transferred to the Eppendorf tubes containing 500-μL isopropanol to precipitate the RNA. RNA was precipitated by mixing and incubating the tubes at room temperature for 10 min and then centrifugation at 12, 000 × g for 10 min. The precipitants were washed once with ethanol 70% and centrifuged at 7500 × g for 5 min. The precipitated RNA was solved in the nuclease-free water, and the concentration was determined using a spectrometer ND-1000 (NanoDrop Technologies, USA).

### 2.8. Real-Time PCR (RT-PCR)

RT-PCR was performed to investigate the expression level of the selected genes in CST-susceptible and -resistant isolates. RT-PCR was done using a One-Step Kappa Biosystem Kit (USA). The materials used in the final volume of 25 μL include 5 μL of RNA with a concentration of 5 ng, 0.5 μL of forward primer, and 0.5 μL of reverse primer with a concentration of 0.15 μM and 12.5 μL of master mix containing KAPA SYBR FAST, which was performed in a Corbett machine in Australia. The temperature program used in RT-PCR included 95°C for 5 min, 95°C for 30 s, 60°C for 30 s, and a final hold at 30°C for 40 cycles. Finally, the relative expression of *pmrA*, *pmrB*, *fliC*, and *agfA* genes was calculated by the ΔΔCт method. The 16S rRNA was used as the housekeeping gene. Used primers for each gene are presented in Table 1.

### 2.9. Statistical Analysis

The SPSS-21 software was used for data analysis. The normalization of differential expression of target genes was defined by REST software. One-way ANOVA was used to analyze the statistical differences among samples.

## 3. Results

### 3.1. Bacterial Isolates

**Figure 1:**
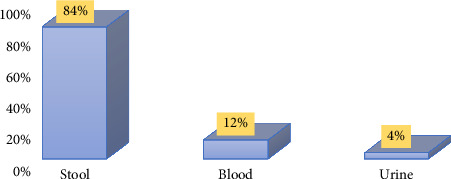
Distribution of samples: 84% (42) of the isolates were acquired from patients' stools, 12% (6) from blood, and 4% (2) from urine.

In this study, 50 *S. enterica* isolates were isolated from Ali Asghar Hospital and Ghadir Mother and Child Hospital. Most isolates (84%) were from stool samples; a low percentage of isolates were from blood and urine samples (12% and 4%, respectively) (Figure 1).

### 3.2. Antibiogram Test

Chi-square test was used to compare the level of sensitivity between six antibiotics. Based on the results of this test, there was a significant difference in the level of sensitivity between six antibiotics (*p* < 0.001). The results of follow-up comparisons revealed that there was no significant difference in sensitivity between the two antibiotics NAL and CFZ (*p* < 0.05) and the sensitivity in these two was significantly lower than the antibiotics CHL, AMP, TET, and CST (*p* > 0.05). There was no significant difference in the sensitivity of the three antibiotics CHL, AMP, and TET (*p* < 0.05), and the sensitivity of these three antibiotics was significantly lower than the sensitivity to CST (*p* < 0.05) (Table 2). As CST is the last-resort treatment option for MDR *S. enterica* since there are few reports of CST-resistant *Salmonella* species in Iran and other countries, the resistance of these bacteria to CST is considered as a failure in an efficient treatment program. In examining the minimum inhibitory concentration for the CST antibiotic, 15% of the isolates were resistant (≥ 4 μg/mL), whereas 85% of the strains were susceptible (≤ 2 μg/mL).

### 3.3. The Results of *hilA* Gene Investigation

**Figure 2:**
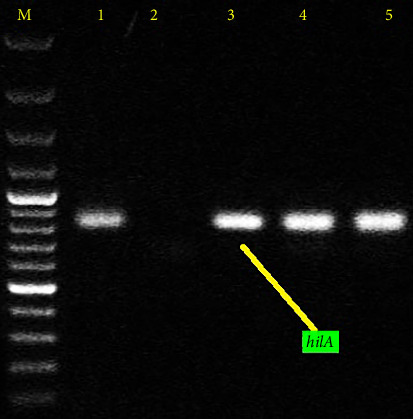
PCR electrophoresis gel of the *hilA* gene, which shows the presence of the 854-bp fragment, which indicates the presence of this gene and confirms the presence of *Salmonella* bacteria. Column M: marker (100 bp), column 1: positive control (*S. typhimurium* ATCC), column 2: negative control (*E. coli* ATCC 14025), and columns 3, 4, and 5: positive samples.

The amplification of the *hilA* gene via PCR revealed an 854-bp band in all samples tested, confirming the presence of *Salmonella* bacteria (Figure 2).

### 3.4. The Results of PCR for Investigating the Presence of *pmrA*, *pmrB*, *fliC*, and *agfA* Genes

**Figure 3:**
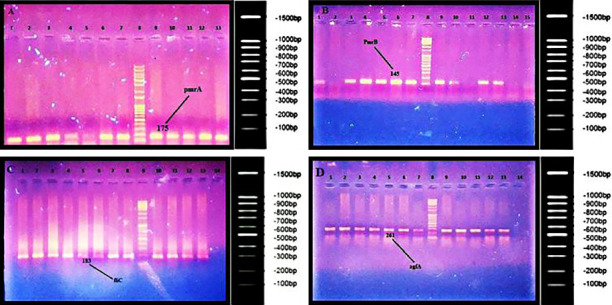
Investigation of *pmrA*, *pmrB*, *fliC*, and *agfA* genes in samples by PCR. In all gels, column 1 was the positive control (DNA template was from *Klebsiella pneumonia* ATCC 700603). (A) All tested samples were *pmrA*^+^ isolates except one (column 5) that was *pmrA*^−^. (B) Out of 15 samples, 11 isolates were *pmrB*^+^ and 4 isolates were *pmrB*^−^. (C) All 13 tested samples were *fliC*^+^. (D) All tested isolates were *agfA*^+^. 1-kb DNA ladder was used in all gels.

**Figure 4:**
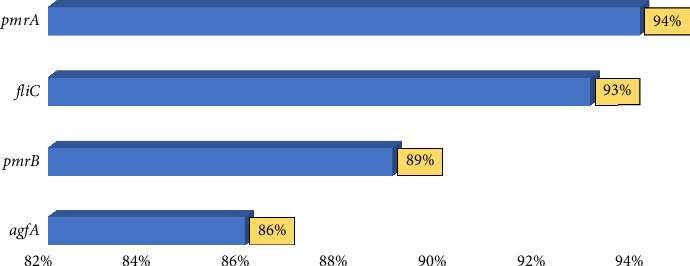
Percentage of *Salmonella enterica* isolates carrying *pmrA*, *pmrB*, *agfA*, and *fliC* genes.

The presence of the selected genes (*pmrA*, *pmrB*, *fliC*, and *agfA*) was investigated using the PCR method. The PCR products were then analyzed using gel electrophoresis (Figures 3(A), 3(B), 3(C), and 3(D)). The 94%, 89%, 93%, and 86% of the isolates carried *pmrA*, *pmrB*, *fliC*, and *agfA* genes, respectively (Figure 4).

### 3.5. Expression Analysis of the Selected Genes Using RT-PCR

**Figure 5:**
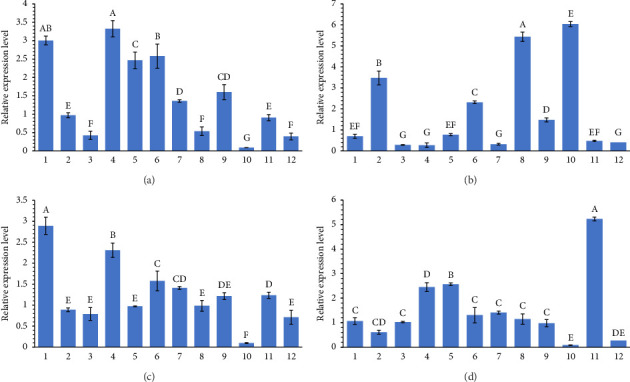
Gene expression changes in the *fliC* (a), *agfA* (b), *pmrA* (c), and *pmrB* (d) genes. In all figures, column 12 represents the expression of 16S rRNA (as internal control); columns 1, 2, and 3 are related to colistin-susceptible isolates, and columns 4, 5, 6, 7, 8, 9, 10, and 11 are related to colistin-resistant isolates.

**Figure 6:**
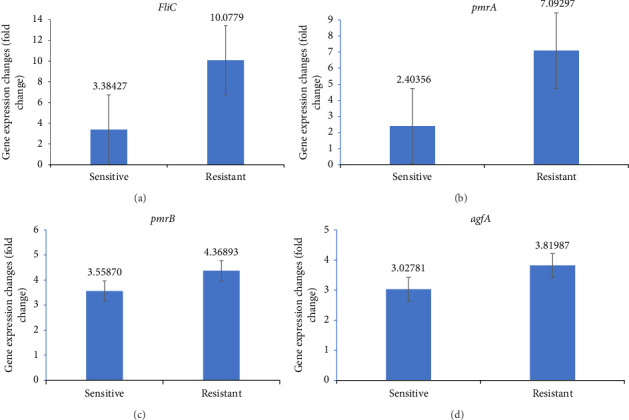
Comparison of fold-chain expression of *pmrA*, *pmrB*, *fliC*, and *agfA* genes in colistin-susceptible and -resistant isolates. One-way ANOVA was used to analyze the statistical differences between samples.

The expression level of *pmrA*, *pmrB*, *fliC*, and *agfA* is presented in Figures 5(a), 5(b), 5(c), and 5(d). The fold-chain expression of all selected genes is presented in Figure 6. As can be seen, the fold-chain expression of all genes was significantly higher in CST-resistant isolates than those in the CST-susceptible ones (*p* < 0.01).

## 4. Discussion

*S. enterica* is the most important cause of salmonellosis in humans, and the prevalence of this infection has increased in recent decades [5]. The emergence of the MDR strains of this bacterium, especially CST-resistant ones, has become a significant health problem. The present study investigated the presence of the CST-resistant *S. enterica* isolates in clinical samples of two hospitals in Shiraz, Iran. Furthermore, the expression level of two CST-resistance associated genes, *pmrA* and *pmrB*, and two virulence genes, *fliC* and *agfA*, in hospital-acquired *S. enterica* samples were studied using RT-PCR.

In 2000, the WHO issued a significant warning about antibiotics referred to as “magic bullets” to end the misfortunes that infections brought to humans [15]. Antibiotic resistance in the community is present among the bacteria that cause primary infections. The community-acquired antibiotic resistance is a serious problem (e.g., resistance to penicillin and macrolide in *Streptococcus pneumonia* and some MDR strains of *Staphylococcus aureus*). The situation of antibiotic resistance in hospitals is more critical than that in the community, which affects the provision of health system services. *S. enterica* are among the bacteria that can acquire resistance to antimicrobial agents in many ways and transfer that trait to the following generations and even to other intestinal bacteria. Accordingly, they can spread and cause many epidemics. Excessive consumption of antibiotics in humans, veterinary, agriculture, and animal food products resulted in the emergence of antibiotic-resistant strains of *S. enterica*, a major epidemiological problem throughout the world. In MDR strains, CST is the last option for the treatment of the infection [21]. It means that the resistance to this antibiotic is a failure in the treatment of *Salmonella*-related diseases. This study indicates that CST resistance is considerable among isolated strains, attributed to improper and excessive consumption of antibiotics [31].

CST exerts its bactericidal activity by binding to bacterial cell membrane and disrupting its permeability, which results in the leakage of intracellular components [26]. Various mechanisms have been proposed for the CST resistance of *S. enterica*. In this bacterium, CST chromosomal resistance includes the activity of two-component regulatory systems (TCRSs) of PmrA/PmrB and PhoP/PhoQ responsible for biosynthetic changes of L-Ara4N and PEtn [34]. Activation of these systems is associated with environmental stimuli, such as the low concentration of Mg2+ or specific mutations in the genes encoding TCRSs. These mutations lead to the practical expression of PmrA/PmrB and PhoP/PhoQ. Plasmid-mediated CST resistance is conferred by MCR genes encoding PEtn [35], which add PEtn to lipid A and, as in chromosomal resistance, and contribute to decreased CST–LPS interactions. Plasmid-mediated resistance is less common in *S. enterica*.

The expression results indicated that the *pmrA* gene expression was significantly higher in CST-resistant strains than that in the susceptible ones. It is well established that the level of the *pmrA* expression is associated with CST-resistant phenotype [36]. However, no significant changes were observed in *pmrB* in CST-resistant and -susceptible strains in the present study. This issue may be due to the high expression level of *pmrB* but not *pmrA* in susceptible strains. In other studies, where the expression levels of *pmrA* and *pmrB* in CST-resistant and CST-susceptible *S. enterica* have been investigated, these two genes had been upregulated. This finding (in this work) may be related to the low number of susceptible isolates.

This study also observed that the flagellin-coding gene, *fliC*, is associated with the CST-resistant phenotype as well. To the authors' best knowledge, it is the first study that investigated the association between the *fliC* expression level of *fliC* and CST-resistant phenotype in *S. enterica*. Bacteria have different molecular identification patterns, such as lipoprotein (peptidoglycan), flagellin, DNA, and RNA, which are identified by various types of toll-like receptors (TLRs) [37]. *Salmonella* flagellin gene *fliC* is detected by TLR5, which activates NFkB to produce inflammatory cytokines such as IL-8, IL-10, pro-IL-1, and pro-IL18. Finally, neutrophils are called to the infection site to eliminate the organism [38]. Accordingly, perhaps mutations in the *fliC* gene cause a change in the structure of bacterial flagellin such that the immune system cannot detect and eliminate the bacterium. This vital issue is associated with increased expression of this gene in CST-resistant strains compared with susceptible ones. This study investigated the association between the *agfA* gene and CST resistance; *agfA* gene is one of the genes encoding the presence of the fimbriae, which also have properties related to pathogenesis and autoaggregation. Still, no significant change was observed in the expression level of the *agfA* gene in resistant and susceptible *S. enterica* strains.

## 5. Conclusions

The findings of this study show the relatively high rate of multidrug- and CST-resistant strains of *S. enterica* in patients, and it is an actual warning for the health community.

The results also showed that CST resistance in *S. enterica* is associated with increased expression of the *pmrA* and *fliC* genes. Still, it is not associated with *pmrB* and *agfA* genes. In the future, the suppression of these genes could contribute to their role in CST resistance and resistance elimination in the treatment process. Future studies can be performed on the suppression of these genes.

## Figures and Tables

**Table 1:** Sequences of primers used in the study.

Primer	Primer sequence	Reference
*pmrA-F*	5′- ATGACAAAAATCTTGATGATTGAAGAT-3′	[31]
*pmrA-R*	5-CCATCATAGGCAATCCTAAATCCA-3	[31]
*pmrB-F*	5-GAACAGCTGAGCACCCTTTAA-3	[31]
*pmrB-R*	5-ACAGGTGGAACCAGCAAATG-3	[31]
*agfA-F*	5-TCCGGCCCGGACTCAACG-3	[32]
*agfA-R*	5-CAGCGCGGCGTTATACCG-3	[32]
*fliC-F*	5-ATAGCCATCTTACCAGTTCCCCC-3	[33]
*fliC-R*	5-GCTGCAACTGTTACAGGATATGCC-3	[33]

**Table 2:** The antibiotics-resistance pattern of *Salmonella enterica* isolates in the present study.

**p** **value**	**Colistin**	**Tetracycline**	**Ampicillin**	**Chloramphenicol**	**Cefazolin**	**Nalidixic acid**	

< 0.001	84% (42)^c^	40% (20)^b^	40% (20)^b^	38% (19)^b^	0% (0)^a^	0% (0)^a^	Sensitive
	16% (8)^c^	60% (30)^b^	60% (30)^b^	62% (31)^b^	100% (50)^a^	100% (50)^a^	Resistant

*Note:* As can be seen, actually all isolates were resistant to nalidixic acid and cefazolin. Antibiotics with signi:cant differences are distinguished with different English letters.

## Data Availability

Data sharing is not applicable to this article as no new data were created or analyzed in this study.

## References

[1] Monte D. F. M., Sellera F. P. (2020). Salmonella. *Emerging Infectious Diseases*.

[2] Banavandi S., Javad M., Shahhosseiny M. H. (2005). Selective Amplification of prt, tyv and invA Genes by Multiplex PCR for Rapid Detection of *Salmonella typhi*. *Iranian Biomedical Journal*.

[3] Kipper D., Hellfeldt R. M., De Carli S. (2019). Salmonella Serotype Assignment by Sequencing Analysis of Intergenic Regions of Ribosomal RNA Operons. *Poultry Science*.

[4] Popa G. L., Popa M. I. (2021). Salmonella Spp. Infection-A Continuous Threat Worldwide. *Germs*.

[5] Rabsch W., Tschäpe H., Bäumler A. J. (2001). Non-typhoidal Salmonellosis: Emerging Problems. *Microbes and Infection*.

[6] Gulig P. A., Curtiss R. (1987). Plasmid-associated Virulence of *Salmonella typhimurium*. *Infection and Immunity*.

[7] Josenhans C., Suerbaum S. (2002). The Role of Motility as a Virulence Factor in Bacteria. *International Journal of Medical Microbiology*.

[8] Milan C., Timm C. D. (2015). Fatores de Virulência Associados à Formação de Biofilme POR *Salmonella enterica*. *Science and Animal Health*.

[9] Yamaguchi T., Toma S., Terahara N. (2020). Structural and Functional Comparison of Salmonella Flagellar Filaments Composed of FljB and FliC. *Biomolecules*.

[10] Hedayat S., Habibi M., Doust H., Reza, Karam A., Reza M. (2022). Design of a Chimeric Protein Composed of FimH, FyuA and CNF-1 Virulence Factors from Uropathogenic *Escherichia coli* and Evaluation its Biological Activity and Immunogenicity In Vitro and In Vivo. *Microbial Pathogenesis*.

[11] Okamura M., Matsumoto W., Seike F. (2012). Efficacy of Soluble Recombinant FliC Protein from *Salmonella enterica* Serovar Enteritidis as a Potential Vaccine Candidate against Homologous Challenge in Chickens. *Avian Diseases*.

[12] Date K. A., Bentsi-Enchill A., Marks F., Fox K. (2015). Typhoid Fever Vaccination Strategies. *Vaccine*.

[13] Edwards R. A., Puente J. L. (1998). Fimbrial Expression in Enteric Bacteria: A Critical Step in Intestinal Pathogenesis. *Trends in Microbiology*.

[14] Aghdasi-Araghinezhad R., Kumarss A. (2017). Study of Antibiotic Resistance Pattern and Incidence of Pathogenic Genes of mgtC, spi4R, agfA, invE/A and ttrC in Salmonella Infantis Isolated From Clinical Specimens. *Feyz Medical Sciences Journal*.

[15] Lushniak B. D. (2014). Antibiotic Resistance: a Public Health Crisis. *Public Health Reports*.

[16] Nelson R. E., Hyun D., Jezek A., Samore M. H. (2022). Mortality, Length of Stay, and Healthcare Costs Associated With Multidrug-Resistant Bacterial Infections Among Elderly Hospitalized Patients in the United States. *Clinical Infectious Diseases*.

[17] Jovčić B., Malešević M., Kojić M. (2022). Genomic Analysis of Multidrug-Resistant *Salmonella enterica* Serovar Kentucky Isolates from Humans, Turkey, and Food in the Republic of Serbia. *Foodborne Pathogens and Disease*.

[18] Glynn M. K., Bopp C., Dewitt W., Dabne P., Mokhtar M. H., Angulo F. J. (1998). Emergence of Multidrug-Resistant *Salmonella enterica* Serotype Typhimurium DT104 Infections in the United States. *New England Journal of Medicine*.

[19] Sherman J. W., Conte J. E. (1987). Ceftriaxone Treatment of Multidrug-Resistant Salmonella Osteomyelitis. *The American Journal of Medicine*.

[20] Nation R. L. (2009). Colistin in the 21st Century. *Current Opinion in Infectious Diasease*.

[21] Sepahvand S., Madani M., Sepahvand H., Davarpanah M. A. (2022). Comparative Assessment of the Mouse Immune Responses to Colistin-Resistant and Colistin-Sensitive Isolates of Acinetobacter Baumannii. *Microbial Pathogenesis*.

[22] Odey T. O. J., Tanimowo W. O., Afolabi K. O., Jahid I. K., Reuben R. C. (2024). Antimicrobial Use and Resistance in Food Animal Production: Food Safety and Associated Concerns in Sub-Saharan Africa. *International Microbiology*.

[23] Boucher H. W., Talbot G. H., Bradley J. S. (2009). Bad Bugs, No Drugs: No ESKAPE! An Update From the Infectious Diseases Society of America. *Clinical Infectious Diseases*.

[24] Sabnis A. S., Hagart K. L., Klöckner A. (2021). Colistin Kills Bacteria by Targeting Lipopolysaccharide in the Cytoplasmic Membrane. *eLife*.

[25] Jovčić B., Novović K., Filipić B. (2020). Genomic Characteristics of Colistin-Resistant *Salmonella enterica* Subsp. Enterica Serovar Infantis From Poultry Farms in the Republic of Serbia. *Antibiotics (Basel)*.

[26] Olaitan A. O., Morand S., Rolain J.-M. (2014). Mechanisms of Polymyxin Resistance: Acquired and Intrinsic Resistance in Bacteria. *Frontiers in Microbiology*.

[27] Gogry F. A., Siddiqui M. T., Sulta I., Haq Q. M. R. (2021). Current Update on Intrinsic and Acquired Colistin Resistance Mechanisms in Bacteria. *Frontiers of Medicine*.

[28] Zahra A., Gholizadeh P., Ganbarov K. (2019). Molecular Mechanisms Related to Colistin Resistance in Enterobacteriaceae. *Infection and Drug Resistance*.

[29] Bertelloni F., Cagnoli G., Turchi B., Ebani V. V. (2022). Low Level of Colistin Resistance and Mcr Genes Presence in Salmonella Spp. *Evaluation of Isolates Collected between 2000 and 2020 from Animals and Environment. Antibiotics (Basel)*.

[30] Bauer A. W., Kirby W. M., Sherris J. C., Turck M. (1966). Antibiotic Susceptibility Testing by a Standardized Single Disk Method. *American Journal of Clinical Pathology*.

[31] Sepahvand S., Davarpanah M. A., Roudgari A., Bahador A., Karbasizade V., Jahromi Z. K. (2017). Molecular Evaluation of Colistin-Resistant Gene Expression Changes in Acinetobacter Baumannii With Real-Time Polymerase Chain Reaction. *Infection and Drug Resistance*.

[32] Freixo R., Albano H., Joana S., Teixeira P. (2011). Case Report of Clinical Salmonellosis by Salmonella Typhimurium that Occurred in Portuguese Children. *Letters in Applied Microbiology*.

[33] Lim Y.-H., Hirose K., Izumiya H. (2003). Multiplex Polymerase Chain Reaction Assay for Selective Detection of *Salmonella enterica* Serovar Typhimurium. *Japanese Journal of Infectious Diseases*.

[34] Lima T., Domingues S., Silva G. J. D. (2019). Lasmid-Mediated Colistin Resistance in *Salmonella enterica*: A Review. *Microorganisms*.

[35] Richter P., Krüger M., Prasad B. (2019). Using Colistin as a Trojan Horse: Inactivation of Gram-Negative Bacteria with Chlorophyllin. *Antibiotics (Basel)*.

[36] Luo Q., Wang Y., Fu H. (2020). Serotype is Associated With High Rate of Colistin Resistance Among Clinical Isolates of Salmonella. *Frontiers in Microbiology*.

[37] Gupta S. K., Bajwa P., Deb R., Chellappa M. M., Dey S. (2014). Lagellin a Toll-Like Receptor 5 Agonist as an Adjuvant in Chicken Vaccines. *Clinical and Vaccine Immunology*.

[38] Broz P., Ohlson M. B., Monack D. M. (2012). Innate Immune Response to *Salmonella typhimurium*, a Model Enteric Pathogen. *Gut Microbes*.

